# Cloud classrooms enhancing continuing medical education during COVID-19 in China

**DOI:** 10.3389/fmed.2023.1174677

**Published:** 2023-04-25

**Authors:** Xi Chen, Taoran Zhang, Jun Li, Xiufen Yang, Lihua Luo, Han He, Yingxiang Huang

**Affiliations:** ^1^Department of Ophthalmology, Beijing Friendship Hospital, Capital Medical University, Beijing, China; ^2^Laboratory Animal Center, School of Life Sciences, Peking University, Beijing, China; ^3^State Key Laboratory of Proteomics, Academy of Military Medical Sciences, Academy of Military Sciences, Beijing, China; ^4^State Key Laboratory of Experimental Hematology, Institute of Hematology, Fifth Medical Center of Chinese People's Liberation Army General Hospital, Beijing, China

**Keywords:** cloud classrooms, COVID-19, continuing medical education (CME), online, medicine

## 1. Introduction

During the COVID-19 pandemic, many measures were taken, including social distancing and isolation in China. Offline academic meetings were largely suspended to prevent spread of COVID-19. Instead, online courses, known as “cloud classrooms,” have been held worldwide (1–4). This resulted in online meetings or courses becoming the main source for physicians to obtain information and improve their knowledge as well as to exchange academic viewpoints, which are an important component of the cloud classroom, especially in China. With advances in Chinese software such as Tencent Meeting, BizConf Video and MCMeet, and other software including Zoom, 100doc, etc., the content of meetings and online courses could be stored online and educational content can be accessed at any time, which comprise the characteristics of a cloud classroom.

The medical profession requires lifelong learning. With advances in technology, continuing medical education (CME) has continuously undergone innovative development. The foundation of innovation is active learning and advances of the Internet have resulted in teaching activities undergoing diversified development in medical education (5). Massive Open Online Courses (MOOC) have been popular in several countries for years to provide education for students who are training to become health care professionals (6–8). For example, the course Clinical Terminology for International and US Students provided by the University of Pittsburgh targets freshmen-level students in the medical field (9). The course Going Out on a Limb: Anatomy of the Upper Limb of the University of Pennsylvania supplements conventional medical education and can even replace traditional lectures such that time spent with the professor can be used for more meaningful discussions. Especially in recent years, there has been a largely increased implementation of MOOCs, which have reached over 220 million learners (40 million new learners in 2021 alone), with over 19,400 courses available, compared with only 35 million learners in 2015 (10).

The COVID-19 pandemic has become a major cause of rapid globalization and digitization for CME projects in China. We have become increasingly more skilled at utilizing the Internet to conduct online academic communication as it is not constrained by region, environment, venue, and traffic. For example, the Brightness Center jointly organized by the Bethune Charitable Foundation and National Clinical Research Center for Eye Diseases of China held 824 online academic meetings in 2022, involving more than 15,000 physicians, which included 12 nationwide meetings. This has provided greater opportunities for more physicians to participate in academic exchange. Details regarding advantages and disadvantages of cloud classrooms in CME warrant further examination. In this perspective paper, we demonstrated the effects of cloud classrooms on CME during the COVID-19 pandemic from the perspective of physicians in China.

## 2. Discussion

### 2.1. Teaching content strengths of cloud classrooms

Cloud classrooms provide a large amount of learning content, different perspectives on diseases, summarized disease characteristics and diagnosis key points, particularly differential diagnosis, and cover research progress nationally and internationally. The cloud classroom has important advantages in terms of scale, openness, and convenience and has become an important educational resource. The cloud classroom also has important effects and a large impact on traditional teaching and provides a brand-new educational option for CME (11–13). Physicians can select content based on their interests, clinical need, or weak areas that require strengthening. It provides greater learning and exchange opportunities for professors to attend more meetings/courses without running around different cities.

Cloud classrooms in China include different types of content, including seminars, special lectures, case discussion, explanation of surgical techniques, surgery video recordings, multidisciplinary consultation meetings, standardized resident physician training, etc. The characteristics of different types of cloud classrooms are as follows.

a. Seminars. These revolve around a theme to be discussed among experts in different hospitals. Consensus can be reached for problems and thoughts can be shared regarding controversial topics. In debates among experts, physicians with different levels of training can be exposed to new ideas regarding clinical diagnosis and treatment and humanistic knowledge in dialectics.
b. Special lectures. An attending physician or professor selects a topic to teach in which they are an expert, and theory and clinical practice experience are included in the course content. These lectures can update understanding on disease diagnosis and treatment, improve diagnosis and treatment levels.
c. Case discussion. The case discussions provide many opportunities for physicians to participate in discussion and make it convenient for departments in different regions to help each other. Case discussions allow one-on-one assistance by clarifying thought processes, highlighting key points in differential diagnosis, and providing solid theoretic knowledge. Diagnosis and treatment levels can be improved by summarizing experiences of treatment outcomes. Especially in China, there are some differences in medical level between developed and underdeveloped cities. By explaining typical or difficult cases, physicians in areas with lower medical level can make faster improvement.
d. Explanation of surgical techniques. Experienced surgical experts show surgery videos for a topic and explain the surgical techniques involved. These can be used as educational materials for young surgeons to learn and improve surgical techniques and are indispensable and valuable resources for surgeon training.
e. Surgery video recordings demonstration. This is a platform for physicians to demonstrate their learning outcomes and where experienced experts can provide critiques to help physicians efficiently improve their surgery competency.
f. Multidisciplinary consultation. Online meetings enable multidisciplinary consultation to be simpler. Different departments do not only discuss the patients' conditions but also an overarching theme, from the perspective of different specialties. This allows physicians to learn about research progress in other specialties. This results in more comprehensive understanding of disease and facilitates coordination between departments.
g. Standardized resident physician training. This is a course designed for inexperienced residents that focuses on foundational knowledge and basic procedures. These are considered to be entry-level courses for residents.

### 2.2. Cloud classrooms stimulate motivation to learn among physicians and promote active over passive learning

Conventional education emphasizes the dominant role of teachers, and students are willing to accept the information provided by teachers. In addition, students often avoid to ask their teacher questions or being asked questions by the teacher/senior physician. Cloud classroom fully encourages enthusiasm and participation among physicians and cultivates independent learning capacity (14, 15). During meetings/courses, physicians can discover their own problems and weaknesses and can identify courses/meetings that are suitable for them in a targeted manner. During courses, physicians can also develop clinical thinking through talks given by lecturers and flexibly using various types of foundational knowledge rather than being constrained by these (16). And also, they are able to use fragments of time to learn and can repeatedly review areas in which they have problems or difficulties (17). This is a change from waiting for knowledge and clinical experience to be imparted by teachers to active learning and personalized learning.

### 2.3. Cloud classrooms make up for differences owing to unbalanced regional development

Regional development can be uneven and levels of health care may differ greatly between regions in China. Regions that are poor and remote have fewer channels for obtaining external information and physicians in these areas have low foreign language proficiency and capacity to read the published literature. Because these regions are remote, transportation is inconvenient and not all physicians are able to attend meetings to acquire the latest medical information owing to constraints of time and venue. Cloud classrooms avoid these problems as courses can be attended online and physicians have opportunities to participate in discussion or receive critiques from well-known professors and achieve one-to-one remote guidance (18). This is important for improving the diagnosis and treatment levels of these physicians. In cloud classrooms held during the COVID-19 pandemic, large-scale hospitals in developed areas have set up assistance teams and provided clinical guidance via the Internet, which has help to improve regional health care levels and decrease differences in health care levels among regions in China.

### 2.4. Shortcomings of cloud classrooms

In comparison with offline face-to-face meetings, there are differences in targeting, affinity and reliability of online learning (19). Face-to-face interactions between people are not limited to lectures and presentations but also involve gestures, postures, and facial expressions. Direct contact tends to improve mutual trust, thereby helping to develop important exchange relationships and allowing strangers to become familiar with each other. Meeting dialogs help deepen friendships and understanding of each other and improves closeness in relationships (20). These are all important interpersonal interactions. Strengthening the ability to communicate with different groups or individuals is also the foundation for becoming an outstanding physician. In face-to-face meetings, the lecturer can adjust the content based on the level of the audience and onsite feedback and can provide more targeted courses. Lecturers can also engage in detailed communication in exchanges outside the course and facilitate questions.

A cloud classroom is rich in content but the viewpoints of lecturers may not be similar. Different viewpoints allow physicians to think for themselves. However, there is a possibility of misjudgment when young physicians hear different viewpoints from different physicians and they may choose to follow a viewpoint that is suitable for their own patients in clinical practice (21). However, “trial and error” is not permitted in clinical practice as injury to patients must be minimized. Hence, tertiary diagnosis and treatment is extremely important in clinical practice. Senior physicians should seek to understand the thinking of junior physicians and promptly correct errors and guide junior physicians to correctly understand the viewpoints of other physicians in a course. Junior physicians should seek advice from senior physicians to avoid errors and make preparations for attempts under the guidance of senior physicians. Therefore, guidance from senior physicians is also required during cloud classroom learning (22).

Cloud classrooms involve an active learning process for physicians but lack of supervision and monitoring. It has been reported that the dropout levels were higher in MOOCs than in offline courses (23). Also, the physician's thirst for knowledge and level of proactivity also affects learning outcomes. Although attendance at meetings may be recorded to ensure that students are online so as to supervise and monitor learning and to ask questions to assess students' learning outcomes, differences between classroom teaching remain. The reliability of and affinity for cloud classrooms are far lower than those of face-to-face exchange.

Cloud classrooms have deficiencies in humanistic education and communication cultivation capacity. Clinical work involves communication with different patients, listening to the hidden meanings behind patients' statements, identifying patients' actual complaints, determining the duration of symptoms from a patient's description, and prompt identification of important positive and negative signs during patient examination, all of which cannot be taught in a cloud classroom (24).

The reliability of the content of cloud classrooms might also be questionable. Cloud classrooms are increasing and their organizers range from societies and associations to well-known large-scale hospitals. The types of meeting range from provincial/municipal meetings and classes at national, municipal, and county levels. All these meetings must have undergone review and approval. Existing units can organize a meeting or class via the collaboration of one or more experts, but a rigorous review system is still lacking. Moreover, information overload and unreliable sources on the Internet can pose a challenge to information acquisition by physicians.

A cloud classroom is highly dependent on network communication. Meeting information can be easily obtained through communities and the Internet in major cities. However, it may still be difficult for physicians in remote cities and hospitals that have lower levels of health services to obtain relevant information, or the information obtained is not as rich as that obtained by physicians in major and mid-level cities. Hence, regional differences are still present. Furthermore, the ease of operation of software affects the selection of audience.

### 2.5. Future expectations

The outbreak of the COVID-19 pandemic caused rapid digitalization of the healthcare industry in teaching and training making it one of the great transformations in medical education. Incorporation of digital approaches such as cloud classrooms in medical training makes healthcare professionals better communicate with less limitations. It is necessary to improve the accessibility of cloud classrooms, which requires developing more intuitive and user-friendly software to achieve a better understanding of user interface. The integration of advanced technical means, such as 5G communication, virtual reality, metaverse and so on, into cloud classrooms is expected to provide a better use of experience, improve educational efficiency and effectiveness (25). On this basis, opening the learning resources and curriculum review function can help the physicians master the learning knowledge better. In the future as social interaction becoming more and more unrestricted, how to combine online and face-to-face approaches more reasonably and make different forms exert the maximum advantage is a problem that all medical educators should consider together.

## 3. Conclusion

Rapid growth in medical technology is immensely changing in medical education, during COVID-19 epidemic. Medical practitioners will be composed of a generation that actively uses digital technology to obtain information instantly. Cloud classrooms will play an important role in achieving a higher level of proficiency by providing more effective and standardized training, providing educators with new roles and designing and promoting better learning experiences with the help of digital technology.

## Author contributions

YH provided the idea and designed the manuscript. XC, TZ, XY, LL, JL, HH, and YH contributed to the conceptualization, writing original draft, and writing—review and editing. All authors contributed to the article and approved the submitted version.
